# Supplementary material for: A molecular mechanism underlying gustatory memory trace for an association in the insular cortex
Source: eLife. 2015 Oct 9;4:e07582. doi: 10.7554/eLife.07582 (PMC4703067; doi:10.7554/eLife.07582)
Supplement: Figure 1—source data 1. — Kruskal-Wallis non-parametric ANOVA (1B, 1E), cluster analysis (1B), Mann-Whitney test (1B) and Friedman's non-parametric repeated measures ANOVA (1C) and independent samples t-test (1F) were conducted to analyse the group effect. DOI: http://dx.doi.org/10.7554/eLife.07582.004 [file elife-07582-fig1-data1.zip › Figure 1source data 1.docx]

**Figure 1- source data 1**

**Figure 1B.** Kruskal-Wallis non-parametric ANOVA: X^2^ (9, n = 157) = 100.862, P < 0.0001; Post-hoc test compared to Control, n = 12; 1h ITI-CTA, n = 10, P < 0.0001; 2h ITI-CTA, n = 32, P < 0.0001; 3h ITI-CTA, n = 18, P < 0.0001; 4h ITI-CTA, n = 10, P = 0.004; 5h ITI-CTA, n = 21, P < 0.001; 6h ITI-CTA, n = 10, P = 0.007; 7h ITI-CTA, n = 11, P = 0.038; 8h ITI-CTA, n = 27, P = 0.029; 20h ITI-CTA, n = 6, P = 0.942.

**Figure 1B.** Cluster Analysis: Cluster1; 1h, 2h, and 3h ITI-CTAs, cluster size: 43.2%. Cluster2: 4h to 8h ITI-CTAs, cluster size: 56.8%. Mann-Whitney test between two clusters, U = 446.00, Z = -8.188, P < 0.0001.

**Figure 1C.** Friedman's non-parametric repeated measures ANOVA: interaction between tests 1, 2, 3, and 4; X^2^ (3, n = 139) = 221.859, P < 0.0001; test1 to test2, X^2^ = 4.785, P < 0.0001; test2 to test3, X^2^ = 5.134, P < 0.001; test3 to test4, X^2^ = 3.995, P < 0.001. Kruskal-Wallis ANOVA on test4; X^2^ (7, n = 139) = 68.212, P < 0.0001. Post-hoc test compared to 1h ITI-CTA; 2h ITI-CTA, P = 0.433; 3h ITI-CTA, P = 0.439; 4h ITI-CTA, P = 0.003; 5h ITI-CTA, P = 0.001; 6h ITI-CTA, P = 0.001; 7h ITI-CTA, P < 0.0001; 8h ITI-CTA, P < 0.0001. Kruskal-Wallis ANOVA on test7; X^2^ (2, n = 30) = 0.601, P = 0.740.

**Figure 1E.** Kruskal-Wallis non-parametric ANOVA: X^2^ (5, n = 48) = 33.186, P < 0.0001; Post-hoc test compared to Control, n = 8; 1h ITI-CTA, n = 8, P < 0.0001; 2h ITI-CTA, n = 8, P < 0.0001; 3h ITI-CTA, n = 8, P = 0.001; 4h ITI-CTA, n = 8, P = 0.027; 5h ITI-CTA, n = 21, P = 0.668;

**Figure 1F**. Independent samples t-test: CTA, n = 5; reverse conditioning, n = 7; T (6.101) = 6.757, P = 0.0004.
