# Supplementary material for: A molecular mechanism underlying gustatory memory trace for an association in the insular cortex
Source: eLife. 2015 Oct 9;4:e07582. doi: 10.7554/eLife.07582 (PMC4703067; doi:10.7554/eLife.07582)
Supplement: Figure 2—source data 1. — Independent samples t-test (2E, 2F, 2G) and one way ANOVA (2I, 2J, 2K) were conducted to analyse the group effect. DOI: http://dx.doi.org/10.7554/eLife.07582.007 [file elife-07582-fig2-data1.zip › Figure 2 source data 1.docx]

**Figure 2- source data 1**

**Figure 2E.** Independent samples t-test: 30min, n = Water, 6 and Novel taste, 6; T (10) = -2.237, P = 0.039; 1h, n = Water, 28 and Novel taste, 28; T (54) = -2.809, P = 0.008; 3h, n = Water, 11 and Novel taste, 11; T (20) = -2.926, P = 0.008; 5h, n = Water, 6 and Novel taste, 6; T (10) = 0.879, P = 0.400; 8h, n = Water, 6 and Novel taste, 6; T (10) = -0.414, P = 0.688.

**Figure 2F.** Independent samples t-test: 30min, n = Water, 6 and Novel taste, 6; T (10) = -0.312, P = 0.762; 1h, n = Water, 22 and Novel taste, 22; T (42) = 1.092, P = 0.286; 3h, n = Water, 11 and Novel taste, 11; T (20) = -0.304, P = 0.765; 5h, n = Water, 6 and Novel taste, 6; T (10) = 0.257, P = 0.803; 8h, n = Water, 6 and Novel taste, 6; T (10) = -0.578, P = 0.570.

**Figure 2G.** Independent samples t-test: 30min, n = Water, 6 and Novel taste, 6; T (10) = -1.268, P = 0.233; 1h, n = Water, 28 and Novel taste, 28; T (54) = -1.062, P = 0.295; 3h, n = Water, 11 and Novel taste, 11; T (20) = -1.441, P = 0.165; 5h, n = Water, 6 and Novel taste, 6; T (10) = 1.095, P = 0.299; 8h, n = Water, 6 and Novel taste, 6; T (10) = -0.555, P = 0.591.

**Figure 2I.** one way ANOVA: n= 14-19 per group, F (2, 51) = 4.736, P = 0.013; post-hoc LSD; water + saline Vs novel taste + saline P = 0.034, novel taste+ saline Vs novel taste + MK801 P = 0.005 and water + saline Vs novel taste + MK801 P = 0.36.

**Figure 2J.** one way ANOVA: F (2, 51) = 0.0290 P = 0.72.

**Figure 2K.** one way ANOVA: F (2, 51) = 0.293, P = 0.747.
