# Supplementary material for: A molecular mechanism underlying gustatory memory trace for an association in the insular cortex
Source: eLife. 2015 Oct 9;4:e07582. doi: 10.7554/eLife.07582 (PMC4703067; doi:10.7554/eLife.07582)
Supplement: Figure 3—source data 1. — Independent samples t-test was conducted to analyse the group effect. The differences between the variances of groups were corrected following Levene’s test for equality of variances. DOI: http://dx.doi.org/10.7554/eLife.07582.012 [file elife-07582-fig3-data1.zip › Figure 3source data 1.docx]

**Figure 3- source data 1**

**Figure 3B.** Independent samples t-test: n = Tatcont,11 and TatCN21, 11; T (20) = 2.302, P = 0.032.

**Figure 3C.** Independent samples t-test: n = Tatcont, 14; TatCN21, 15; T (27) = 2.061, P = 0.049.

**Figure 3D.** Independent samples t-test: n = Tatcont, 24 and TatCN21, 25; T (47) = 0.251, P = 0.803.

**Figure 3E.** Independent samples t-test: n = Tatcont, 11; TatCN21, 14; T (23) = 1.248, P = 0.225.
