# Supplementary material for: A molecular mechanism underlying gustatory memory trace for an association in the insular cortex
Source: eLife. 2015 Oct 9;4:e07582. doi: 10.7554/eLife.07582 (PMC4703067; doi:10.7554/eLife.07582)
Supplement: Figure 4—source data 1. — Independent samples t-test (4A, 4C, 4D, 4E, 4F, 4G) was conducted to analyse the group effect. Pearson’s correlation was used to analyse the association between the association between GluA1 and pT286CaMKIIα (4B). DOI: http://dx.doi.org/10.7554/eLife.07582.018 [file elife-07582-fig4-data1.zip › Figure 4 source data 1.docx]

**Figure 4- source data 1**

**Figure 4A.** GluA1/β-Tubulin; n = water, 37 and novel taste, 38; T (61.256) = -3.982, P = 0.0001. pS831GluA1/GluA1; T (73) = -0.117, P = 0.907.

**Figure 4B.** Pearson correlation: water, R = 0.120, P = 0.478 and novel taste, R = 0.370, P = 0.020.

**Figure 4C.** n = Water+ Tatcont, 9, Novel taste+ Tatcont, 10, Novel taste+ TatCN21 10; between Tatcont injected water and novel taste; T (12.117) = -2.773, P = 0.014. between Tatcont+water and TatCN21+novel taste T (17) = -0.806, P = 0.431.

**Figure 4D.** n = saline, 11 and CNQX, 14; T (14.363) = 3.275, P = 0.005.

**Figure 4E.** n= saline, 10 and CNQX, 12; T (14.55) = 2.09, P = 0.041.

**Figure 4F.** n = saline, 9 and CNQX, 9; T (16) = 2.227, P = 0.049.

**Figure 4G.** n = saline, 13 and CNQX, 13; T (24) = 0.136, P = 0.893.
